# Porous Materials from Thermally Activated Kaolinite: Preparation, Characterization and Application

**DOI:** 10.3390/ma10060647

**Published:** 2017-06-12

**Authors:** Jun Luo, Tao Jiang, Guanghui Li, Zhiwei Peng, Mingjun Rao, Yuanbo Zhang

**Affiliations:** School of Minerals Processing & Bioengineering, Central South University, Changsha 410083, China; luojun2013@csu.edu.cn (J.L.); jiangtao@csu.edu.cn (T.J.); zwpeng@csu.edu.cn (Z.P.); raomingjun2003@126.com (M.R.); sintering@csu.edu.cn (Y.Z.)

**Keywords:** porous material, kaolinite, thermoactivation, adsorption, heavy metal ions

## Abstract

In the present study, porous alumina/silica materials were prepared by selective leaching of silicon/aluminum constituents from thermal-activated kaolinite in inorganic acid or alkali liquor. The correlations between the characteristics of the prepared porous materials and the dissolution properties of activated kaolinite were also investigated. The results show that the specific surface area (SSA) of porous alumina/silica increases with silica/alumina dissolution, but without marked change of the BJH pore size. Furthermore, change in pore volume is more dependent on activation temperature. The porous alumina and silica obtained from alkali leaching of kaolinite activated at 1150 °C for 15 min and acid leaching of kaolinite activated at 850 °C for 15 min are mesoporous, with SSAs, BJH pore sizes and pore volumes of 55.8 m^2^/g and 280.3 m^2^/g, 6.06 nm and 3.06 nm, 0.1455 mL/g and 0.1945 mL/g, respectively. According to the adsorption tests, porous alumina has superior adsorption capacities for Cu^2+^, Pb^2+^ and Cd^2+^ compared with porous silica and activated carbon. The maximum capacities of porous alumina for Cu^2+^, Pb^2+^ and Cd^2+^ are 134 mg/g, 183 mg/g and 195 mg/g, respectively, at 30 °C.

## 1. Introduction

Due to their high toxicity and degradation resistance, accumulated heavy metal pollutants cause serious damage in aquatic and soil systems, and in the human body [1,2,3]. The pollution of aquatic environments, especially caused by heavy metals, is a problem of worldwide concern. The heavy metal ions in wastewater can be adsorbed by using porous materials with a fluffy structure and special functional groups [4,5]. The main adsorbents include activated carbon and porous mineral materials [6,7,8]. Activated carbon has the advantages of simple preparation, high efficiency and recyclability, despite its exorbitant price and short lifespan [4,5,7].

The layered aluminosilicates with high specific surface area and superior ion exchange ability can be satisfactorily used as mineral adsorption materials [9,10,11]. Currently, many researchers have been focusing on the exploitation of porous materials from natural-layered clays for adsorption of heavy metal ions, such as Cu(II), Pb(II), Cd(II) and Cr(III), Cr(VI) etc. [9,11,12]. The development of pillared interlayer clay (PILC) is based on exchangeable and expandable layered clays [13,14]. The PILC materials with high pore volume, tunable pore size and ordered structure can be prepared by inserting specific ions or ion-clusters in the interlayer of layered clays [14,15]. They are widely used in the fields of catalysis, selective adsorption and environmental protection [15,16].

To improve the adsorption capacities of layered clays, mechanical/thermal activation processes were performed to create the active sites and increase the surface area of clays [11,17]. Stefanova [17] has reported that a modified clay marl is obtained by thermal activation at 750 °C, and this prepared product can be successfully used for removal of metal ions from water solutions in a wide range of concentrations. However, the adsorption capacities of modified clays are highly dependent on the activation temperature. If the temperature increases to some extent, the adsorption increases, while if the clay is activated at higher temperatures, the adsorption properties of modified clays may be decreased [11]. This is mainly caused by the crystal structure transformation during activation.

Clay modified via inorganic acid treatment has also been exploited to remove heavy metal ions from aqueous solutions [18,19,20]. The surface area, porous structure and acidity of the clays can be improved after acid leaching [19]. Furthermore, acid treatments can replace exchangeable cations with H^+^ ions and Al^3+^ in tetrahedral and octahedral sites, resulting in the improvement of adsorption capacities [20]. However, the efficiency of treatments depends on the type of clays, exchangeable cations, and acid; as well as the treatment temperature and time [21]. It means that the restrictions on the scope of acid treatment become severe. For these reasons, mechanical/thermal activation followed by a selective leaching process has been developed further [11,22,23,24,25,26,27,28,29]. The clays first undergo mechanical or thermal treatment to activate silica or alumina before selective leaching [24,25,26,27,28,29]. This process enhances the dissolution of alumina/silica constituents, the pores are created on the surface of granules and between the layered microstructures after leaching, resulting in a superior surface area [24,26,28,29]. Okada et al. [24] reported that the porous silica material with specific surface area of 340 m^2^/g can be obtained by acid leaching of the activated kaolinite (roasted at 600 °C for 24 h) in 20% H_2_SO_4_ liquor at 90 °C for 0.5–5 h. Furthermore, the ions exchangeability of kaolinites modified via thermal roasting-acid leaching is superior to kaolinites modified only by thermal activation [22].

Obviously, mechanical/thermal activation followed by selective leaching is a more efficient process for the preparation of porous materials from natural layered clays than single acid modification or mechanical/thermal treatment. The adsorption properties of modified clays are closely correlated with dissolution of alumina/silica, which depends on their activation level. In this study, based on the premise of investigation of silica and alumina activation after roasting of natural kaolinite mineral, the porous alumina and silica materials were prepared separately from thermally activated kaolinite by selective alkali or acid leaching, and the correlations between their properties, including specific surface area, pore size and pore volume, and silica/alumina dissolution of kaolinite activated at various temperatures, were investigated. Lastly, the adsorption capacities of prepared porous materials for the heavy metal cations including Cu^2+^, Cd^2+^ and Pb^2+^ were evaluated.

## 2. Materials and Methods

### 2.1. Materials

**Figure 1:**
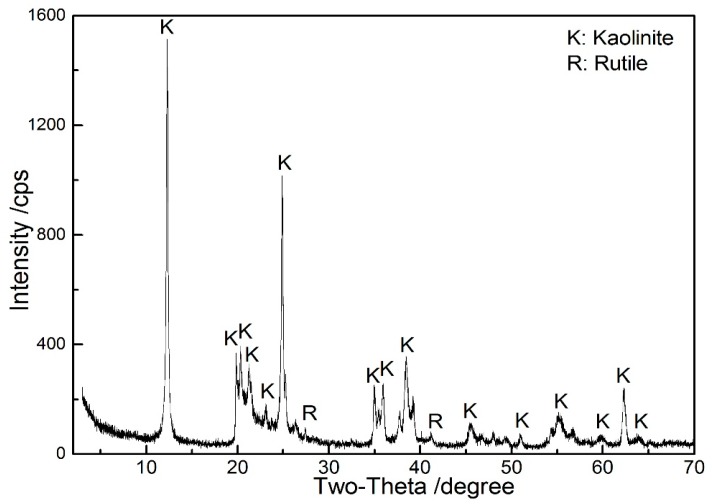
XRD pattern of kaolinite raw ore.

Natural kaolinite raw ore with a purity of 91% was taken from the Guangdong province of China. From Table 1, the Al_2_O_3_ and SiO_2_ contents were 38.8% and 42.2%, respectively. The XRD result in Figure 1 confirms that kaolinite is the predominant mineral, and the majority TiO_2_-bearing impurity is rutile. Prior to the experiments, the kaolinite samples were ground to 70% of the particle size below 150 μm.

Leaching reagents (HuiHong Reagent, Changsha, China), including sulfuric acid (H_2_SO_4_) and sodium hydroxide (NaOH), used in this work were of analytical grade. Additionally, copper chloride (CuCl_2_), cadmium sulfate (CdSO_4_) and lead nitrate (Pb(NO_3_)_2_) of analytic grade were used to prepare the aqueous solutions for adsorption tests.

### 2.2. Methods

#### 2.2.1. Preparation of Porous Materials

Thermal activation followed by selective leaching was performed for the preparation of the porous alumina/silica materials. Thermal activation was carried out in an electric-heating furnace (U-Therm, Changsha, China) with dimension of 50 cm × 20 cm × 18 cm. The pulverized kaolinite sample was loaded into the corundum plates (6 cm × 3 cm) and heated isothermally at given temperatures for 15 min. Then the activated products were cooled to room temperature for the subsequent leaching tests. At the beginning of the leaching trial, the aqueous leaching solution (sulfuric acid or caustic soda liquor) and activated products were added to pots equipped with Teflon protective lining. The sealed pots were soaked in a glycerine bath and rotated at 30 rpm (for a schematic of the reactor equipment refer to [30]). The acid leaching tests were performed at 120 °C for 90 min in 20% H_2_SO_4_ liquor with liquid to solid ratio (L/S) of 10:1 mL/g, and the dissolution of silica was performed at 120 °C for 2 h in 120 g/L NaOH liquor with L/S of 10:1 mL/g. Filtration was performed immediately after leaching, and the residues, namely porous materials, were collected and dried.

#### 2.2.2. Adsorption Tests

In the adsorption tests, the various aqueous solutions were independently prepared by dissolving the reagents including CuCl_2_, CdSO_4_ and Pb(NO_3_)_2_ in the distilled water. The initial concentrations of Cu^2+^, Cd^2+^ and Pb^2+^ are 50 mg/L, 50 mg/L and 100 mg/L, respectively. The quantified adsorbent (1.0 g/L of dose for Cu^2+^ adsorption and 0.4 g/L of dose for Cd^2+^ and Pb^2+^ adsorption) and aqueous solution containing heavy metal ions were simultaneously added into the flask, which was oscillated in an oven controlled crystal oscillator at a temperature of 30 °C for a time of 60 min. The agitation speed was kept at 160 rpm. The solid-liquid separation was performed after adsorption via a centrifuge, and the heavy metal cations in the supernatant were measured. Based on the concentration of heavy metal cations in the simulated wastewater and supernatant, the removal efficiency of the adsorbent for heavy metal ions was calculated.

#### 2.2.3. Instrumental Techniques

The X-ray diffraction patterns of samples were obtained by using an XRD (D/Max 2500; Rigaku, Tokyo, Japan) with a copper Kα X-ray source with the scanning angle ranging from 3° to 65° (2θ) in 0.02° (2θ) steps at scanning speed of 8°/min.

Specific surface area (SSA) is an important index for assessing the adsorption capacity of porous materials. The SSA of porous materials was determined by using the Brunauer-Emmett-Teller (BET) method (QuadraSorb SI, Quantachrome Instruments, Boynton Beach, FL, USA). The pore size distribution of porous materials was also determined based on the Barret-Joyner-Halenda (BJH) method.

The microstructures of porous materials were characterized by a scanning electron microscope (SEM; JSM-6360LV, JEOL, Tokyo, Japan). SEM images were recorded in the backscattered electron mode operating under a low vacuum condition (0.5 Torr and 25 keV). The powder was coated with a thin layer of gold prior to the detection. A transmission electron microscope (TEM; JEM-3010, JEOL, Tokyo, Japan) was also used for characterization of microstructures of porous silica materials.

ICP-OES spectrometry (IRIS Intrepid II XSP, Thermo Elemental, Waltham, MA, USA) was used to measure the concentrations of heavy metal cations in the supernatant after adsorption.

## 3. Results and Discussion

### 3.1. Preparation of Porous Materials

**Figure 2:**
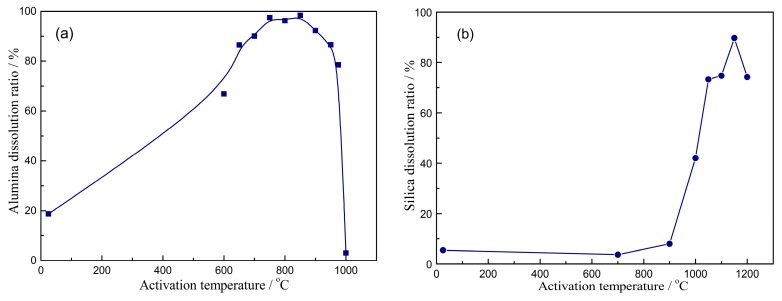
Effects of activation temperature on dissolution of alumina (**a**) and silica (**b**).

During the thermal activation, the phase formation and physicochemical properties of kaolinite are altered as the temperature increases [31,32,33]. As a result, the dissolution of silica and alumina from activated kaolinite is also different. It can be seen in Figure 2 that the alumina/silica dissolution from activated kaolinite increases initially and then decreases with the increase of activation temperature. For dissolution of alumina from activated kaolinite in sulfuric acid liquor (seen in Figure 2a), only 18.7% alumina is dissolved from raw kaolinite without activation. This value is markedly increased when the activation temperature is in the range from 650 °C to 950 °C. However, when the temperature increases from 850 °C to 1000 °C, the dissolution ratio of alumina decreases from 98.4% to 3.1%. For dissolution of silica from activated kaolinite in alkali liquor (in Figure 2b), the dissolution ratio remains at a lower level of about 8%, when the temperature is below 900 °C. When the temperature increases to 1000–1200 °C, the dissolution of silica is improved and the value reaches 89.7% at 1150 °C for 15 min.

**Figure 3:**
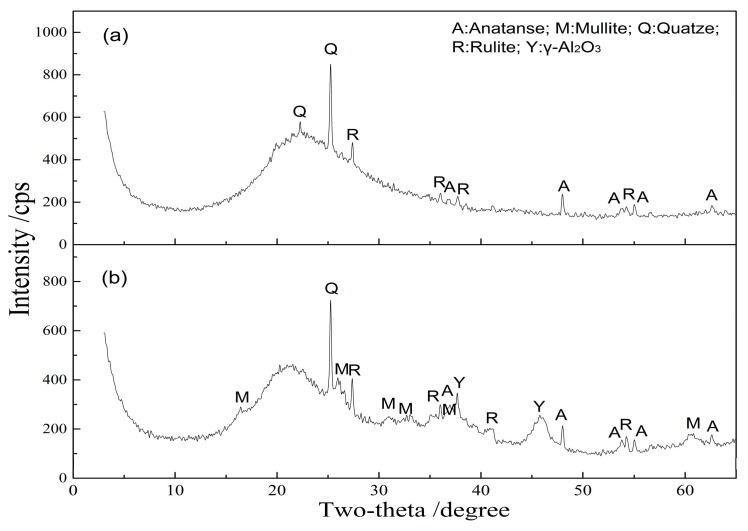
XRD patterns of kaolinite activated at 900 °C for 15 min (**a**) and at 1150 °C for 15 min (**b**).

**Figure 4:**
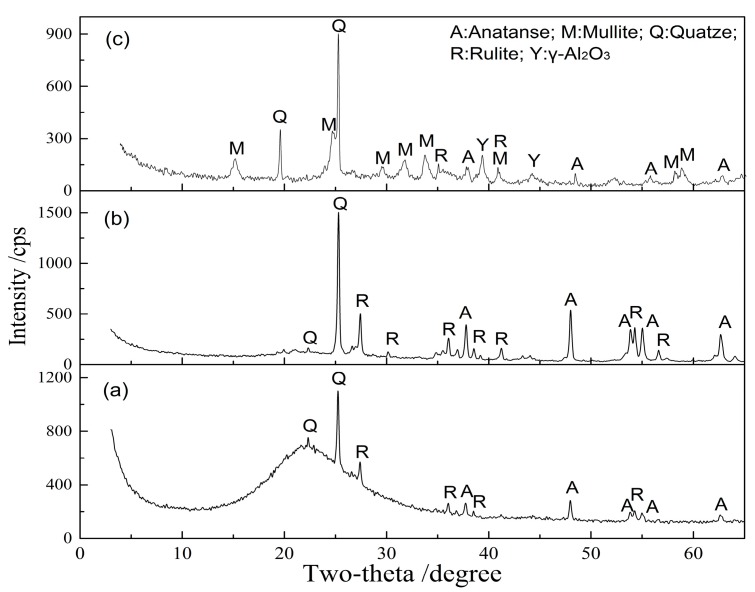
XRD patterns of leaching residues from activated kaolinite (**a**) residue from acid leaching of kaolinite activated at 900 °C for 15 min; (**b**) residue from alkali leaching of acid-leached residue; (**c**) residue from alkali leaching of kaolinite activated at 1150 °C for 15 min.

From Figure 2, the appropriate activation temperatures for alumina and silica are different. The XRD patterns of kaolinite activated at 900 °C and 1150 °C for 15 min are shown in Figure 3. Compared with the raw kaolinite ore (Figure 1), the peaks of kaolinite disappear in the activated kaolinite, meanwhile, a wide and asymmetrical diffraction peak belonging to amorphous metakaolin in the range of 15°–30° 2θ appears in kaolinite activated at 900 °C (Figure 3a) [32,33]. After acid leaching, the main phases in acid leaching residue have no significant change (Figure 4a). However, the type of amorphous phase may be different due to leaching of the aluminum constituent. To verify this change, the acid-leached residue was further leached in 120 g/L alkali solution at 120 °C for 2 h with L/S of 10:1 mL/g. The results show that 93.3% of the silica is dissolved, while the value is only 8.0% during alkali leaching of kaolinite activated at 900 °C (Figure 2b). Figure 4b also confirms that the amorphous phase disappears after alkali leaching. It means that the Al-O-Si bond in metakaolin obtained by activation roasting of kaolinite is broken during acid leaching, and the defected Si-O tetrahedron is in favor of silica dissolution. As a consequence, the main phases of acid-leached residue are amorphous silica and quartz.

The XRD result in Figure 3b also presents an amorphous peak at 15°–30° 2θ appears in kaolinite activated at 1150 °C for 15 min. It means that the primary silicon in the kaolinite is converted into amorphous silica after roasting at 1150 °C [32]. Due to the amorphous silica with superior alkali leaching behavior [32,34,35], satisfying silica removal can be obtained after alkali leaching of activated kaolinite under suitable thermal treatment (Figure 2b). The XRD result in Figure 4c confirms that the main phases of alkali-leached residue are γ-Al_2_O_3_, mullite and quartz. The insoluble mullite and quartz hinder the dissolution of silica from activated kaolinite and about 10% silica in kaolinite activated at 1150 °C for 15 min is not dissolved after alkali leaching (Figure 2b).

Based on the above analyses, the generation of amorphous metakaolin and amorphous silica under appropriate activation temperatures is characterized by the superior acid dissolution of alumina and alkali dissolution of silica. It means that the porous alumina/silica materials can be prepared by selective leaching of silica or alumina from activated kaolinite. The characteristics of prepared porous materials significantly impact on the dissolution of alumina/silica during leaching process. Hence, the prepared porous alumina and silica materials need firstly to be characterized.

### 3.2. Characterization of Porous Materials

#### 3.2.1. Specific Surface Area

**Figure 5:**
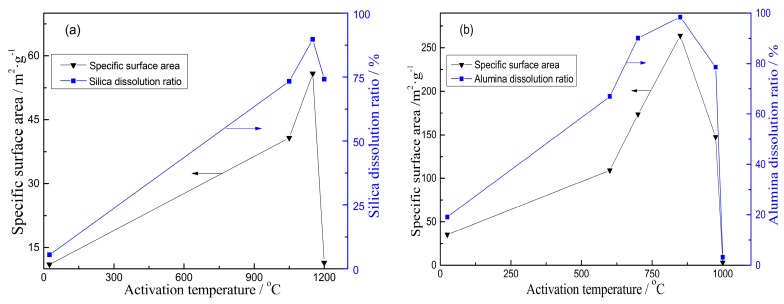
Correlation between the SSA of porous materials and dissolution of activated kaolinite. (**a**) Porous alumina; (**b**) porous silica.

The correlations between the SSA of prepared porous alumina/silica materials and silica/alumina dissolution of activated kaolinite are shown in Figure 5. It can be seen in Figure 5a that the silica dissolution ratio of raw kaolinite without thermal activation is only 5.5%. The corresponding SSA of porous alumina is 11.0 m^2^/g. There is no change, when compared with raw kaolinite’s SSA of 10.9 m^2^/g. With an increase in activation temperature, the SSA of porous alumina initially increases with the silica dissolution. When the raw kaolinite is activated at 1150 °C, the silica dissolution of activated kaolinite goes up to 89.7%, and the SSA of porous alumina increases to a maximum of 55.8 m^2^/g. However, at 1200 °C, the silica dissolution of activated kaolinite decreases to 74.2%, and the SSA drops to 11.4 m^2^/g, accordingly.

As seen in Figure 5b, the alumina dissolution ratio of raw kaolinite without activation is 18.7%, with an SSA of 35.3 m^2^/g after acid leaching. With increasing activation temperature, the SSA of porous silica shares the same tendency of alumina dissolution. Its value attains a maximum of 280.3 m^2^/g when the alumina dissolution is 98.4% for kaolinite roasted at 850 °C for 15 min. This result shows that the SSA of porous silica is much higher than that of porous alumina. By further increasing the activation temperature to 1000 °C, the alumina dissolution ratio decreases to 3.1%, and the SSA decreases to 3.0 m^2^/g.

#### 3.2.2. Pore Size

**Figure 6:**
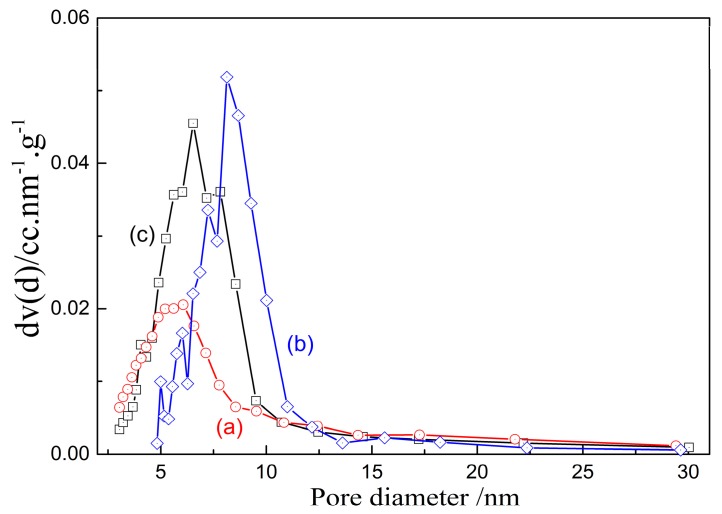
BJH pore size distributions of porous alumina. (Porous alumina prepared via alkali leaching of kaolinite activated at (**a**) 1150 °C; (**b**) 1050 °C and (**c**) 900 °C, for 15 min.

With the dissolution of silicon or aluminum constituents, the pore size and their distribution in porous materials also changes. To determine the correlation between pore size of prepared porous alumina and silica dissolution of activated kaolinite, the pore size distributions of porous alumina obtained from alkali leaching of kaolinite roasted at 900 °C, 1050 °C and 1150 °C for 15 min were considered, and the results are shown in Figure 6 and Table 2, respectively. There is only a single peak in each distribution curve for porous alumina materials. Most pores are in the size range of 5–10 nm, indicating the mesoporous structures of the prepared porous alumina (Figure 6). The BJH pore sizes of various porous alumina are also similar. After alkali leaching of the kaolinite activated at 1150 °C for 15 min, the BJH pore size is 6.06 nm and the value is slightly lower than that of porous alumina. The pore volume of porous alumina decreases as the silica dissolution of activated kaolinite increases (in Table 2). The pore volume of porous alumina prepared from kaolinite activated at 1150 °C is 0.1455 mL·g^−1^.

**Figure 7:**
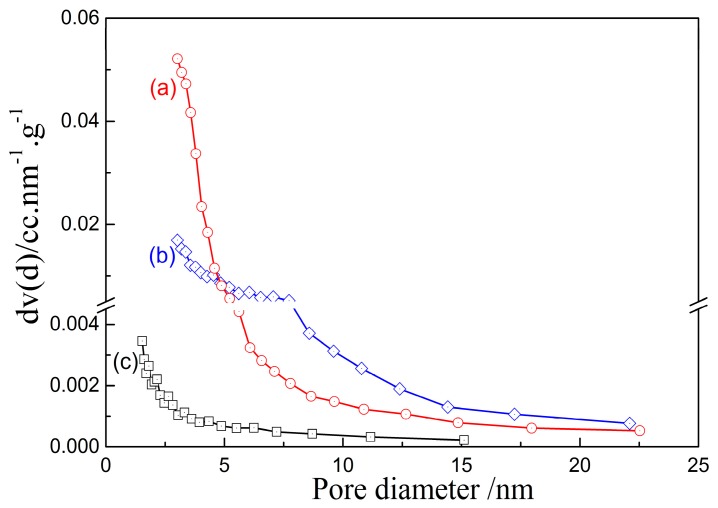
BJH pore size distributions of porous silica (porous silica prepared via acid leaching of kaolinite activated at (**a**) 975 °C; (**b**) 850 °C and (**c**) 600 °C, for 15 min.

The pore size distributions of porous silica prepared from acid leaching of activated kaolinite are also presented in Figure 7, which confirms that the pore size distributions of various porous silica materials are also similar, despite the size distributions of pores becoming smaller as the activation temperature increases, and the pore size being distributed more uniformly with decreasing temperature. As seen in Figure 7, most pores are less than 5 nm in size, which are smaller than those of porous alumina. A large number of mesoporous and micro-pores result in the great specific surface area (Figure 5b). The BJH pore size of porous silica increases when the activation temperature increases, while the pore volume presents an opposite trend (in Table 3). When the raw kaolinite ore is activated at 850 °C for 15 min, the prepared porous silica material has a BJH pore size of 3.05 nm and pore volume of 0.1945 mL·g^−1^ (Table 3).

#### 3.2.3. Microstructure

**Figure 8:**
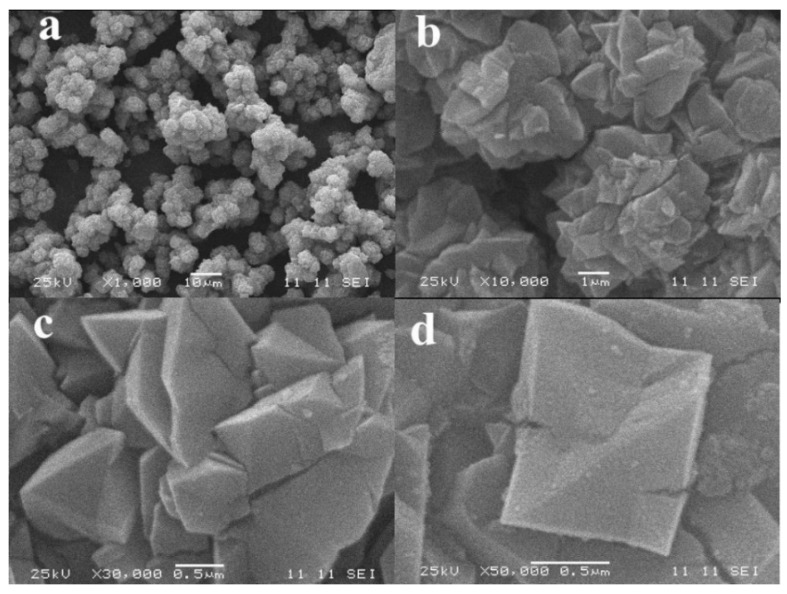
SEM images of porous alumina prepared via alkali leaching of kaolinite activated at 1150 °C for 15 min. (**a**) ×1000; (**b**) ×10,000; (**a**) ×30,000; (**b**) ×50,000.

The microstructure of porous alumina prepared from alkali leaching of kaolinite activated at 1150 °C for 15 min was obtained. As seen from Figure 8a the size of porous alumina material via alkali leaching has an evident decrease compared with kaolinite raw ore. The alumina granules with a size of about 5 μm agglomerate and form spheres with diameters of 10–20 μm. These microstructures of porous alumina are consistent with conventional porous materials. Figure 8b,c shows that a large number of pores with size of 0.5–1 μm exist between the alumina granules. These granules present a multifaceted structure with an irregular spinel surface, and there are cracks and interspaces on their surfaces (Figure 8c,d).

**Figure 9:**
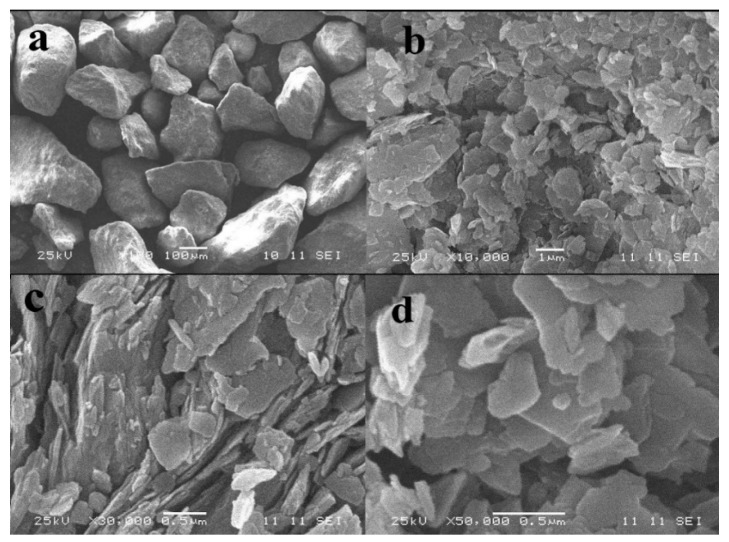
SEM images of porous silica prepared via acid leaching of kaolinite activated at 850 °C for 15 min. (**a**) ×100; (**b**) ×10,000; (**c**) ×30,000; (**b**) ×50,000.

**Figure 10:**
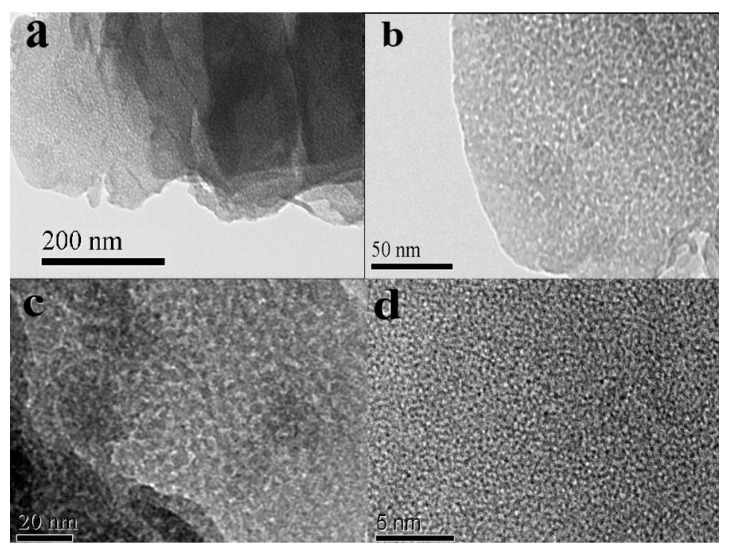
TEM (**a**,**b**) and HRTEM images (**c**,**d**) of porous silica prepared via acid leaching of kaolinite activated at 850 °C for 15 min.

The morphology of porous silica was also observed. It can be seen in Figure 9 that the profiles of porous silica granules and porous alumina granules (Figure 8) are completely different. The particles of porous silica remain small lumps with sizes of 100–150 μm obtained by the superposition of platelets with diameters of 1–3 μm and thicknesses of 10–20 nm. The results indicate that the size of granules has no obvious change after leaching, while the silica platelets are injured. Figure 9b–d also shows that a large number of pores with sizes of below 1 μm exist between the silica platelets. For further examination of the pore structure of silica platelets, TEM and HRTEM images were measured by using a transmission electron microscope. The results in Figure 10 confirm that the laminated structure of particles with a large number of shallow-hole micropores remains in the prepared porous silica material, resulting in the large specific surface area (Figure 5b).

### 3.3. Adsorption of Cu^2+^, Cd^2+^ and Pb^2+^ in Aqueous Solution Using Porous Materials

**Figure 11:**
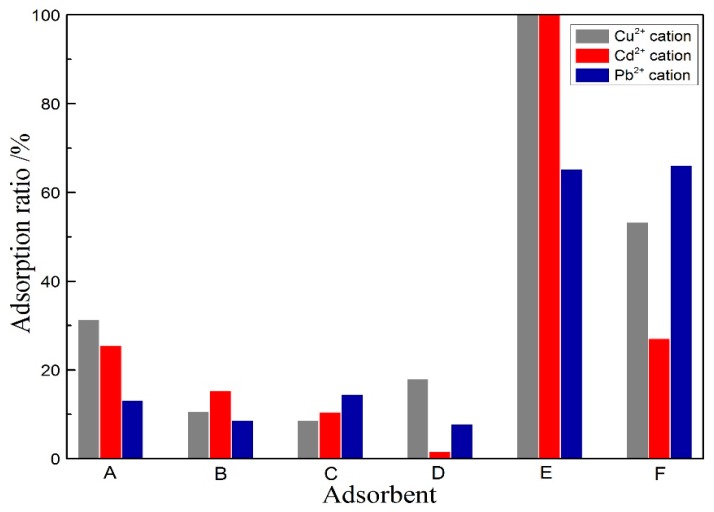
Adsorption capabilities for Cu^2+^, Cd^2+^ and Pb^2+^ on various adsorbents.

The above results indicate that the porous alumina and silica materials with high activity and large specific surface area can be prepared from natural kaolinite ore. The adsorption properties of prepared porous materials for heavy metal, including Cu^2+^, Cd^2+^ and Pb^2+^, were investigated further, and some other samples were used for comparison. The comparative adsorbents are listed in Table 4, and the adsorption results are shown in Figure 11.

It can be seen in Figure 11 that the porous alumina material (E) possess satisfactory adsorption capacity for Cu^2+^, Cd^2+^ and Pb^2+^; while the other adsorbents, including raw kaolinite, activated kaolinite and porous silica material, are inferior under the same adsorption conditions. The removal efficiencies of porous alumina for Cu^2+^, Cd^2+^ and Pb^2+^ are 100%, 99.9% and 65.3%, respectively; while the removal efficiencies of porous silica are only 8.66%, 10.5% and 14.5%, respectively. For comparison, the removal efficiencies of activated carbon for Cu^2+^ and Cd^2+^ are 53.3% and 27.1%, respectively. The results show that the adsorption capacities of porous alumina for Cu^2+^ and Cd^2+^ are much higher than those of activated carbon with a specific surface area of 450 m^2^/g. The approximate adsorption capacity of porous alumina material and activated carbon for Pb^2+^ is also displayed. The saturated adsorption tests further indicate that the maximum capacities of prepared porous alumina material for Cu^2+^, Pb^2+^ and Cd^2+^ are 134 mg/g, 183 mg/g and 195 mg/g, respectively, at 30 °C.

Generally, the major factors affecting adsorption include cation exchange capacity, surface charge, specific surface area, and specific surface structure of adsorbents; as well as pH value of aqueous solution, adsorption time and temperature, etc. [11,18,36]. Compared with the porous alumina material, the adsorption capacities of porous silica for Cu^2+^, Cd^2+^ and Pb^2+^ are unsatisfactory, in spite of the larger specific surface area. The spherical alumina granules with an irregular diamond surface are much more effective for adsorption of heavy metal ions than plate-like silica granules. Besides, there exist a large number of –Al–OH groups on the surface of alumina sheet due to hydroxylation in aqueous solution [22]. The heavy metal ions can be adsorbed or precipitated on the surface of porous alumina granules by its reaction with hydroxyl groups [18]. Lastly, the dissolution of silica may induce a charge imbalance that causes the introduction of cations to achieve the charge balance. The larger pore size and pore volume may also contribute to the adsorption of heavy metals.

## 4. Conclusions

The preparation of porous alumina/silica materials via selective leaching of silica or alumina from activated kaolinite resulted from the generation of metakaolin or amorphous silica along with superior acid dissolution of alumina or alkali dissolution of silica, respectively, under the appropriate activation temperatures.

(1) The characterization of prepared porous materials indicates that their specific surface area (SSA) increases with the increasing dissolution of alumina/silica in aqueous leaching solution from activated kaolinite. The pore volume of porous alumina decreases with the increasing dissolution of silica, while there is no correlation between pore volume of porous silica and alumina dissolution of activated kaolinite. The prepared porous alumina/silica materials belong to the mesoporous materials, despite the slight change of their pore size with the dissolution of activated kaolinite.
(2) When the porous alumina is obtained via alkali leaching of kaolinite activated at 1150 °C for 15 min, the SSA, BJH pore size and pore volume are 55.8 m^2^/g, 6.06 nm and 0.1455 mL/g, respectively. For the porous silica prepared via acid leaching of kaolinite activated at 850 °C for 15 min, these values are 280.3 m^2^/g, 3.06 nm and 0.1945 mL/g, respectively. The adsorption tests confirm that the prepared porous alumina has a superior adsorption of Cu^2+^, Pb^2+^ and Cd^2+^, with maximums of 134 mg/g, 183 mg/g and 195 mg/g, respectively. However, the porous silica has difficulty adsorbing the above-mentioned heavy metal ions.

## Figures and Tables

**Table 1:** Chemical composition of kaolinite raw ore/%.

Al_2_O_3_	SiO_2_	Fe_2_O_3_	TiO_2_	CaO	MgO	K_2_O	Na_2_O	LOI ^1^
38.8	42.2	0.5	4.0	0.2	0.1	0.1	0.3	13.7

^1^ LOI: loss on ignition.

**Table 2:** Correlation between the pore features of porous alumina and silica dissolution of activated kaolinite.

Activation Temperature/°C	Silica Dissolution/%	BJH Pore Size/nm	Pore Volume/mL·g^−1^
900	8.9	6.54	0.2221
1050	73.3	6.57	0.2215
1150	89.7	6.06	0.1455

**Table 3:** Correlation between the pore features of porous silica and alumina dissolution of activated kaolinite.

Activation Temperature/°C	Alumina Dissolution/%	BJH Pore Size/nm	Pore Volume/mL·g^−1^
600	67	1.53	0.1963
850	98	3.05	0.1945
975	79	3.06	0.1313

**Table 4:** Various adsorbents used in the comparative adsorption experiments.

Number	Adsorbent
A	Raw kaolinite ore
B	Activated kaolinite at 850 °C for 15 min
C	Porous silica prepared via acid leaching of activated kaolinite at 850 °C for 15 min
D	Activated kaolinite at 1150 °C for 15 min
E	Porous alumina prepared via alkali leaching of activated kaolinite at 1150 °C for 15 min
F	Activated carbon
